# Detecting vision loss in intermediate age-related macular degeneration: A comparison of visual function tests

**DOI:** 10.1371/journal.pone.0231748

**Published:** 2020-04-16

**Authors:** Susanne G. Pondorfer, Manuel Heinemann, Maximilian W. M. Wintergerst, Maximilian Pfau, Annika L. Strömer, Frank G. Holz, Robert P. Finger

**Affiliations:** Department of Ophthalmology, University of Bonn, Bonn, Germany; Eye Hospital, Charité, GERMANY

## Abstract

The purpose of the study was to evaluate the diagnostic accuracy of visual function tests in intermediate age-related macular degeneration (iAMD). A total of 62 subjects (38 patients with iAMD and 24 controls) were included and underwent several functional assessments: Best-corrected visual acuity (BCVA), low luminance visual acuity (LLVA), visual acuity (VA) measured with the Moorfields Vanishing Optotypes Acuity Charts (MAC), contrast sensitivity with the Pelli-Robson test, reading speed using the International Reading Speed texts (IReST) and mesopic and dark-adapted microperimetry (S-MAIA, CenterVue, Padova, Italy). Groups were compared using non-parametric Wilcoxon rank sum tests and ROC analyses. Linear regression was used to control for confounding. Results showed that all visual function test performances except the IReST were significantly reduced in iAMD patients compared to controls (p < 0.05). These effects did not alter after controlling for age and sex. Best discrimination between iAMD and controls yield the combination of LLVA and contrast sensitivity as well as MAC-VA and contrast sensitivity (ROC area under the curve 0.95 and 0.93, respectively). Our results suggest that LLVA, MAC-VA, contrast sensitivity and mesopic and dark-adapted microperimetry can capture visual impairment characteristic for iAMD. Best discrimination against iAMD is achieved with a combination of two tests.

## Introduction

Age-related macular degeneration (AMD) is the leading cause of visual impairment in the elderly in industrialized countries and an important public health problem [1,2]. Approximately 30–50 million people are affected by AMD worldwide [3]. In Europe 24.1% of people aged over 60 are affected with early or intermediate stages and 2.2% suffer from late AMD [4]. Due to current demographic trends, AMD is expected to increase considerably in the future [3,4].

Early stages of the disease are usually not associated with visual symptoms and patients typically perform well in conventional visual function tests under high luminance and high contrast. Nevertheless, patients may report difficulties and vision loss under low lighting, low contrast and changing light conditions [5–7]. These symptoms may occur even in the earliest stages of AMD when best-corrected visual acuity (BCVA) is unaffected [5,8]. However, the most widely used outcome measure in ophthalmic research is BCVA [9,10] measured with a high-contrast high-luminance chart with single black optotypes on a white background, such as the Early Treatment Diabetic Retinopathy Study (ETDRS) chart. These conventional charts appear to be largely insensitive to the specific functional impairment in early and intermediate AMD and to monitor disease progression [11–13]. Hence, more sensitive visual function tests are required [10,8].

The functional deficit under reduced luminance and/or contrast has been well documented in patients with early and intermediate AMD [14,15] using a number of different functional assessments such as low luminance visual acuity (LLVA) [8,14,16–18], visual acuity (VA) measurements with the Moorfields Vanishing Optotypes Acuity Chart (MAC), which employs high-pass filtered letters [19], contrast sensitivity tests [20–23,15,24,16,25], and fundus-controlled perimetry [12,26–28,9,9,29]. Combinations of any of these visual function tests might further increase sensitivity to detect changes in visual function in particular in early stages of AMD.

However, to date no study has employed all visual function tests previously identified as sensitive to the specific functional impairment in intermediate AMD (iAMD) and compared their ability to discriminate between iAMD and healthy controls. This, however, is required in order to inform selection of the best test or combination of tests in future observational or interventional studies assessing functional impairment in iAMD. Thus, we evaluated and compared an extensive battery of functional tests in patients with iAMD and in healthy controls.

## Methods

We conducted a cross-sectional study at the Department of Ophthalmology, University of Bonn, Germany, from January 2017 until August 2018. The study was approved by the Institutional Review Board of the University Bonn (approval ID: 013/16). Written informed consent was obtained from all participants. The protocol followed the tenets of the Declaration of Helsinki.

38 patients with iAMD and 24 healthy subjects were recruited from the AMD outpatient clinic, the self-help organisation Pro Retina and family members of the patients. As common with exploratory studies, no formal sample size calculation was done, as to date no information is available on the test-retest reliability of all function tests or the responsiveness to change of the measures.

Inclusion criteria for the AMD group were drusen > 125 μm and/or any AMD pigmentary abnormalities according to the classification system introduced by Ferris et al. [30]. For the control group inclusion criteria was BCVA of 20/20. Exclusion criteria for both groups were age <50 years, the presence of choroidal neovascularization, geographic atrophy (GA) or nascent GA [31], significant lens opacity, any corneal pathology that could compromise vision, amblyopia, diabetes, glaucoma, neurological or systemic disease affecting vision, refractive errors >6.00 dioptres (D) of spherical equivalent and >2.00 dioptres (D) of astigmatism. One eye of each patient (the one with the better visual acuity) was included in the study. If both eyes fitted the inclusion criteria and had the same visual acuity, the right eye was chosen. In addition to the functional tests spectral domain optical coherence tomography was performed using a 25° x 25° scan field (49 B-scans, automated real-time mode 20 frames, centred on the fovea) as well as fundus autofluorescence and infrared confocal scanning laser ophthalmoscopy (all with Spectralis OCT2, Heidelberg Engineering, Heidelberg, Germany) and objective refraction measurement using an autorefractor (ARK-560A; Nidek, Gamagori, Japan). All patients also underwent a clinical examination including dilated funduscopy.

### Visual function tests

The following visual function tests were included: BCVA using ETDRS charts, LLVA, BCVA using MAC charts, contrast sensitivity measurement using Pelli-Robson charts, reading speed using the International Reading Speed texts (IReST) as well as mesopic and dark-adapted microperimetry using the modified MAIA “microperimeter” (S-MAIA, CenterVue, Padova, Italy). Visual acuity and functional tests were performed before fundus imaging to avoid bleaching. Patients wore their best correction for all tests except for microperimetry. BCVA was assessed according to the EDTRS method [32] at a testing distance of 4 m. The charts were installed in a standard light box and a subjective refraction was performed prior testing based on the values from the Nidek autorefracor. BCVA was performed with the room lights off and windows covered. The light box was illuminated with two cool daylight 20 watt fluorescent tubes. When the light box was turned on and room lights are off, background illumination of the chart was approximately 150 cd/m^2^. Charts 1 and 2 were used for right and left eyes respectively. To measure LLVA was measured at the same distance and with the same ETDRS charts but with a 2.0-log unit neutral density filter placed in front of the study eye that reduces luminance by 100 fold, leading to an illumination of the chart of 1.5 cd/m^2^, which is in the mesopic range of vision [17]. VA measurement with the MAC charts was also performed at 4 m distance and according to EDTRS method. The MAC charts were of identical layout as the ETDRS charts except that they employed a high-contrast, high-pass letter design with a grey background of the same mean luminance as the letters. Detection and recognition threshold for these letters are almost identical under foveal viewing conditions in normal subjects. After the resolution limit is reached the letters seem to disappear, which is why the test is also called “vanishing optotypes” [19,33].

Contrast sensitivity was measured using the Pelli-Robson charts presented at 1 m distance. The charts consist of 16 triplets of 4.9 x 4.9 cm letters. Contrast decreases by a factor of 0.15 log units in each successive triplet, reading from left to right [34,35,20]. The mean chart luminance was 85 cm/m^2^. VA reached in the prior described visual function tests was expressed in the number of letters read. For BCVA, LLVA and MAC testing, 85 letters correspond to logMAR 0.0, and five letters correspond to one logMAR unit (i.e. 90 letters = logMAR -0.1). For the Pelli Robson charts, three letters correspond to 0.0 log contrast sensitivity unit (CS) and the maximum of 48 letters to 2.25 log CS.

To assess reading speed the IReST was used, which consists of standardized text paragraphs. Texts were presented at a viewing distance of 40 cm and size of 10-point Times New Roman font, which corresponds to normal newspaper print size [36]. Patients wore their best near correction and were asked to read one paragraph aloud while they were timed with a stopwatch. Errors and skip of words were counted and subtracted from the total word count to compute corrected reading in words / minute according to the following formula (correctly read words/reading speed [in seconds] x 60) [36]. All tests were performed monocularly with the other eye covered with an eye-patch.

Mesopic and dark-adapted microperimetry were then performed after pupil dilation with 1.0% tropicamide. Macular sensitivity was measured using the modified S-MAIA device, which performs fundus tracking using a line-scanning laser ophthalmoscope with a super-luminescent diode illumination with a central wave light of 850 nm. For mesopic testing, the standard white LED of the device was used and for dark-adapted red testing the additional red LED (627 nm) was used to project the stimuli. As previously described a customized stimulus grid was used that consisted of 33 points located at 0°, 1°, 3°, 5° and 7° from fixation [37]. All patients underwent mesopic and dark-adapted microperimetry. First mesopic testing was performed, where patients were not dark-adapted and the room light was switched off just before the examination. After mesopic testing all patients underwent 30 minutes of dark adaptation while waiting in the examination room (light level < 0.1 lx). The microperimetric results were summarized as mean sensitivity (MS) in dB.

### Statistical analysis

Statistical comparisons were performed using two-sided significance tests. Baseline demographic and clinical variables were summarized for each group. Due to the sample size most results were not normally distributed (tested for with the Shapiro-Wilk test) so that non-parametric tests were used for analysis. Pairwise differences were calculated using the non-parametric Wilcoxon rank sum test. A p-value < 0.05 was considered statistically significant. To ensure that the findings were not confounded by different demographic characteristics across groups, simple linear regression was performed controlling for age and sex. For each visual function test and for all combination of tests accuracy was assessed using receiver operating characteristics (ROC) curve analysis and equality of the area under the ROC curves (AUCs) of different tests and combination of tests was investigated [38]. A ROC curve plots the sensitivity against the false-positive rate (1-specificity) in which each point reflects values obtained at a different cut-off value from–in this case–a continuous measure. The trade-off between sensitivity and specificity can be visualised on the ROC curve as the cut-off is shifted [39]. The calculation of AUC allows comparison of discriminative ability among the different functional tests. AUC values range from 0.5 to 1.0, where 1.0 represents perfect ability to discriminate between patients with AMD and patients without AMD and 0.5 represents the discrimination resulting from pure chance [40]. An AUC greater than 0.9 is considered excellent, greater than 0.8 to 0.9 very good, 0.6 to 0.7 average and < 0.6 poor [41]. In addition to the ROC curves the Youden index and optimal cut-off point was determined for each visual function test. The Youden index is a commonly used measure of overall diagnostic effectiveness. The index rages between 0 and 1, with values close to 1 indicating better effectiveness and values close to 0 indicating limited effectiveness [42,43]. The cut-point that achieves this maximum is referred to as the optimal cut-point because it is the cut-point that optimizes the visual function tests’ differentiating ability when equal weight is given to sensitivity and specificity [44]. As the functional tests are presented in different units, cut-points for combined tests cannot be reported.

Statistical analyses were performed using the statistical software STATA [45].

## Results

A total of 62 participants were included in the study; 38 patients with iAMD (69.1 ± 7.5 years, range 50–84, 68.4% female), and 24 controls (61.7 ± 6.1 years, range 50–73, 58.3% female). All patients underwent all study assessments. Patients’ characteristics are summarized in Table 1. Control patients were significantly younger than patients with iAMD (p < 0.05).

**Table 1:** Characteristics of patients with intermediate AMD (iAMD) and Controls.

Characteristics	iAMD	Controls	P-value
			iAMD vs Controls
**Mean Age (SD)** ^**a**^**; range, in years**	69.3 (7.5); 50–84	61.7 (6.1); 50–73	< 0.05
**Patients, n**	38	24	
**Women, n (%)**	26 (68.4)	14 (58.3)	0.419
**Men, n (%)**	12 (31.6)	10 (41.7)	

^a^ SD = standard deviation

### Functional test measures between groups

**Fig 1:**
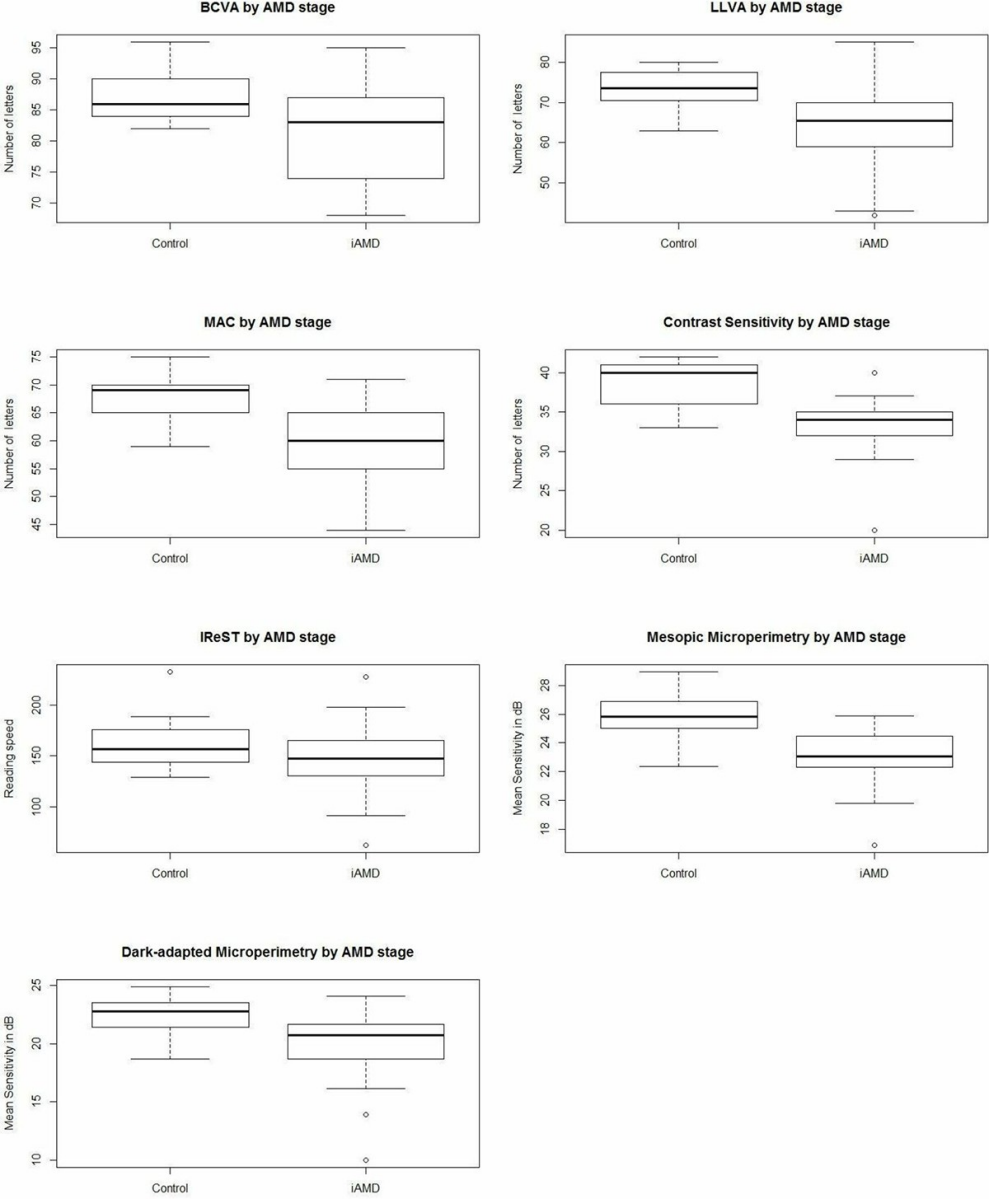
Visual function tests in controls and intermediate AMD.

All performed visual function tests measures are given in Table 2. Boxplots in Fig 1 show the distribution over all functional tests over the two groups. All visual function test performances except the IReST were significantly decreased in the iAMD group compared to controls (p < 0.05). These effects did not alter after controlling for age and sex (Table 2).

**Table 2:** Visual function tests measures: Descriptive analysis and group comparisons.

Functional Test	Statistic	Intermediate AMD	Control group	P-value^a^ iAMD vs Controls	Adjusted P-value^b^ iAMD vs Controls
**BCVA (letters)**	Mean (SD) Min, Median, Max	81,6 (7.2) 68; 83; 95	87.3 (3.9) 82; 86; 96	**< 0.01**	**< 0.01**
**LLVA (letters)**	Mean (SD) Min, Median, Max	63.7 (9.7) 42; 65.5; 85	73.4 (4.5) 63; 73.5; 80	**< 0.01**	**< 0.01**
**MAC (letters)**	Mean (SD) Min, Median, Max	59.9 (6.8) 44; 60; 71	68.1 (4.2) 59; 69; 75	**< 0.01**	**< 0.01**
**Pelli Robson (letters)**	Mean (SD) Min, Median, Max	33.3 (3.4) 20; 34; 40	38.8 (2.9) 33; 40; 42	**< 0.01**	**< 0.01**
**IReST (Reading speed = [words/minute])**	Mean (SD) Min, Median, Max	147.4 (29.8) 62; 147.5; 228	162.3 (23.1) 129; 157; 233	**0.06**	0.445
**Mesopic Microperimetry (dB)**	Mean (SD) Min, Median, Max	23.1 (1.8) 17; 23; 26	25.9 (1.6) 22; 26; 29	**< 0.01**	**< 0.01**
**Dark-adapted Microperimetry (dB)**	Mean (SD) Min, Median, Max	20.0 (2.7) 10; 21; 24	22.5 (1.5) 19; 23; 25	**< 0.01**	**< 0.01**

^**a**^ P-values based on the Wilcoxon rank sum test, SD = standard deviation, BCVA = best-corrected visual acuity, LLVA = low luminance visual acuity, MAC = Moorfields Vanishing Optotypes Acuity Charts (MAC), Pelli Robson = Pelli-Robson contrast sensitivity test, IReST = International Reading Speed Text

^**b**^ Ordinary least squares regression, adjusted for age and sex

Boxplots showing best-corrected visual acuity (BCVA), low luminance visual acuity (LLVA), visual acuity measured with the Moorfields Vanishing Optotypes Acuity Charts (MAC), contrast sensitivity measured with the Pelli-Robson contrast sensitivity test, reading speed measured with the International Reading Speed Text (IReST), mesopic and dark-adapted microperimetry for controls and intermediate AMD (iAMD). Each boxplot includes the maximum (upper whisker), upper quartile (top of the box), median (horizontal line in box), lower quartile (bottom of the box) and minimum (lower whisker) values.

### ROC analysis

**Fig 2:**
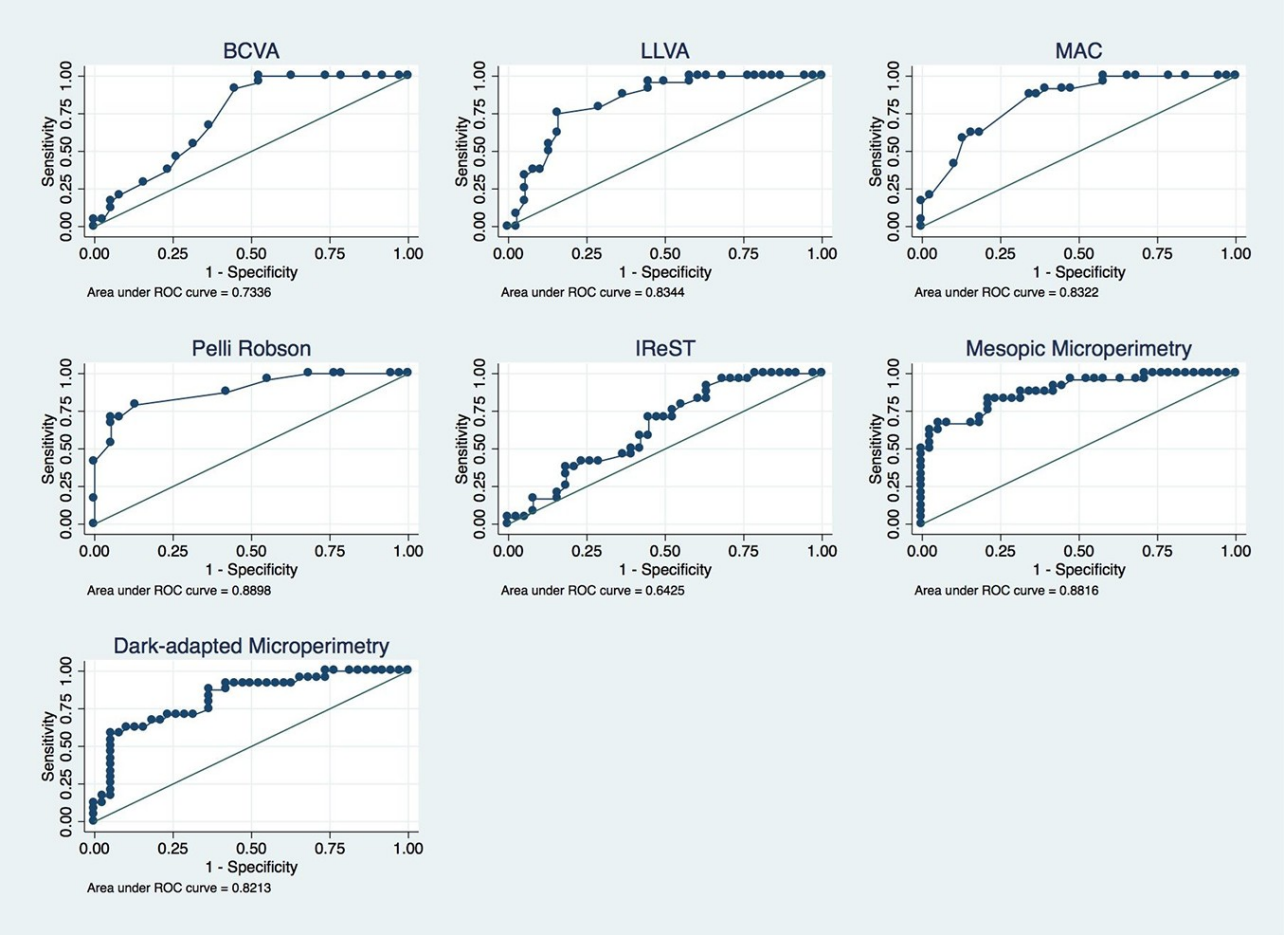
ROC curves of visual function tests.

**Fig 3:**
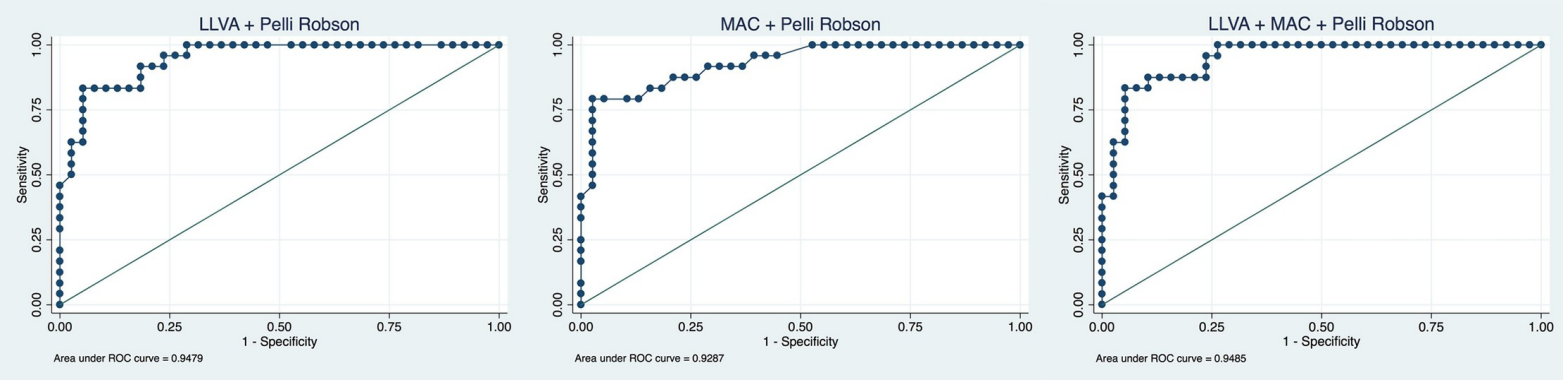
Combined ROC curves.

The AUC values and Youden index for each visual function test are shown in Table 3. Fig 2 depicts ROC curves for all 7 visual function tests. The ROC curve for the Pelli-Robson test is closest to perfect discrimination and yields the best AUC with 0.89 and Youden index with 0.66 followed by mesopic microperimetry (AUC = 0.88, Youden index = 0.62). LLVA and MAC have equal AUC values (0.83), but LLVA has a slightly higher Youden index (0.59 vs. 0.53) followed by dark-adapted microperimetry (AUC = 0.82, Youden index = 0.53) and BCVA (AUC = 0.73, Youden index = 0.47). The ROC curve of the IReST is closest to the reference line and has an AUC of 0.64 and a Youden index of 0.28. Combined ROC analysis showed best results for the combination of LLVA and the Pelli-Robson test (AUC = 0.95), which is significantly higher than the AUCs of all single function test expect mesopic microperimetry. The MAC in combination with the Pelli-Robson test yields an AUC of 0.93. A combination of more than two tests does not improve the results, i.e. combining LLVA, MAC and the Pelli-Robson test yields an AUC of 0.95 as well (Fig 3). All other possible combinations yielded lower AUC values and were therefore not reported.

**Table 3:** AUC values, standard errors, 95% confidence intervals, Youden Index and optimal cut-point.

Functional Test	AUC	Standard error	95% Confidence Interval	Youden Index	Optimal cut-point
**BCVA**	0.73	0.06	0.61–0.85	0.474	81 (letters)
**LLVA**	0.83	0.05	0.73–0.93	0.592	70.5 (letters)
**MAC**	0.83	0.05	0.73–0.93	0.533	64.5 (letters)
**Pelli Robson**	0.89	0.04	0.81–0.97	0.660	35.5 (letters)
**IReST**	0.64	0.07	0.51–0.77	0.285	136 (words/minute)
**Mesopic Microperimetry**	0.88	0.04	0.79–0.96	0.623	24.78 (dB)
**Dark-adapted Microperimetry**	0.82	0.05	0.71–0.92	0.531	22.45 (dB)
**LLVA + Pelli Robson**	0.95	0.02	0.89–0.99		
**MAC + Pelli Robson**	0.93	0.03	0.86–0.99		
**LLVA + MAC + Pelli Robson**	0.95	0.03	0.89–0.99		

BCVA = best-corrected visual acuity, LLVA = low luminance visual acuity, MAC = Moorfields Vanishing Optotypes Acuity Charts (MAC), Pelli Robson = Pelli-Robson contrast sensitivity test, IReST = International Reading Speed Text

ROC curves of best-corrected visual acuity (BCVA), low luminance VA (LLVA), Moorfields Vanishing Optotypes Acuity Charts (MAC), Pelli-Robson contrast sensitivity test, reading speed with the International Reading Speed Text (IReST), mesopic and dark-adapted microperimetry. The ROC curves plots the sensitivity against the false-positive rate (1 –specificity) in which each point reflects values obtained at a different cutoff value from a continuous measure. The diagonal black line serves as a reference line since it is the ROC curve of a diagnostic test that randomly classifies the condition.

Combined ROC curves of low luminance visual acuity (LLVA) with Pelli-Robson contrast sensitivity test, combined Moorfields Vanishing Optotypes Charts (MAC) with Pelli-Robson contrast sensitivity test and all three tests combined (LLVA, MAC and Pelli Robson). The diagonal black line serves as a reference line since it is the ROC curve of a diagnostic test that randomly classifies the condition.

## Discussion

In this study we found visual function tests of central retinal function under low luminance and low contrast conditions to be most impacted in iAMD. Discriminating between iAMD patients and controls a combination of two simple functional tests (e.g. LLVA and Pelli Robson) yielded best results comparable to mesopic microperimetry. Thus, a combination of visual functional tests under low luminance and challenging contrast conditions seems to be the functional assessment best suited to the specific functional impairment in iAMD.

Our findings regarding BCVA and LLVA are comparable with previous studies, which reported a decreased visual function in these tests in iAMD compared to controls [46,16,8]. Wu and colleagues found BCVA, LLVA and mesopic microperimetry significantly reduced for all AMD groups except early AMD compared to controls which is in keeping with our study [14]. Chandramohan and coworkers [8] did not find significant differences for contrast sensitivity between the two groups. This discrepancy to our results is likely a due to different contrast sensitivity tests: Chandramohan and colleagues used a computerized test whereas we used Pelli Robson charts. Earlier studies which also used the Pelli Robson test are comparable to our results [20,20,24,16].

In our study we also included the recently developed MAC charts. Shah et al. first demonstrated the MAC chart’s ability to detect functional loss due to AMD when BCVA tested with EDTRS charts still was unaffected [19]. We could reproduce these findings in our study as we found significant differences in MAC-VA between iAMD and controls. Reading performance assessed with the IReST test was unable to differentiate between iAMD and controls. This in contrast to the findings from Varadaraj et al [47] who demonstrated that AMD patients read slower than controls when forced to read out loud. However, their study included participants with late AMD.

Although BCVA differed significantly in iAMD patients compared to controls, its AUC value was lower compared to all other function test except the IReST. Both, mesopic and dark-adapted microperimetry revealed reduced retinal sensitivity in iAMD patients compared to controls. These results are in accordance with findings from previous studies, which found mesopic microperimetry to be a good functional test in iAMD [20,48,49,12,14,37,8,50,5]. Contrary to our findings Nebbioso and colleagues reported a reduction in scotopic sensitivity but not mesopic sensitivity in patients with hard drusen [51]. This likely is explained by the different study populations. In our sample of more advanced AMD mesopic microperimetry seemed to be a better test compared to dark-adapted microperimetry. This may be attributable to the higher variability of dark-adapted microperimetry compared to mesopic microperimetry [37].

Strengths of our study include the large number of visual function tests assessed including the relatively new MAC charts for which little data are available to date. Additional to several visual acuity and contrast sensitivity tests we also performed mesopic and dark-adapted microperimetry. Additional strengths are the extensive phenotyping and staging of participants using comprehensive retinal imaging in addition to a clinical assessment as well as the statistical exploration of combination of tests for better discrimination. The study is limited by the relatively small sample size as well as a lack of longitudinal data. Another limitation is the fact, that controls were significantly younger than patients. It is conceivable that a subset of eyes classified as “healthy” using the Beckman Classification is affected by pre-clinical AMD. For example, Owsley et al. could demonstrate that impaired dark-adaptation in apparently “healthy” eyes is associated with the incidence of AMD 3 years later [52]. Moreover, Sauer et al. revealed that a subset of elderly “healthy” eyes shows subtle signatures indicative of AMD in fluorescence lifetime imaging ophthalmoscopy [53]. Accordingly, additional longitudinal follow-up will be needed to evaluate the genuine performance of these functional tests as intended for example by the MACUSTAR consortium [54]. In the absence of longitudinal data, however, employed means for classification are in accordance with current gold standards and published literature. With no longitudinal data, we also cannot comment on the predictive value of these tests. As common with exploratory studies, no adjustment for multiple testing was done which might lead to an over-estimation of statistical power. However, ROC analyses is unaffected by multiple testing which makes it unlikely that our findings are purely spurious.

## Conclusions

In our study a combination of tests of central retinal function under low luminance and challenging contrast conditions seem to best capture the specific functional impairment in iAMD. These tests should be explored in longitudinal studies as to both their ability to discriminate between different AMD stages as well as to predict progression.
